# Tumor treating fields therapy is feasible and safe in a 3-year-old patient with diffuse midline glioma H3K27M — a case report

**DOI:** 10.1007/s00381-022-05465-z

**Published:** 2022-02-18

**Authors:** Hanna Gött, Silke Kiez, Hildegard Dohmen, Malgorzata Kolodziej, Marco Stein

**Affiliations:** 1 Department of Neurosurgery, University Hospital Giessen, Giessen, Germany; 2 Department of Pediatric Hematooncology, University Hospital Regensburg, Regensburg, Germany; 3 Institute of Neuropathology, University Hospital Giessen, Giessen, Germany

**Keywords:** Glioblastoma, Glioma, Diffuse midline glioma, TTFields, Pediatric glioma

## Abstract

Since high grade gliomas are aggressive brain tumors, intensive search for new treatment options is ongoing. For adult patients with newly diagnosed (ndGBM) and recurrent glioblastoma (rGBM), low intensity intermediate frequency alternating electric fields, known as tumor treating fields (TTFields) have been established as a new treatment modality. Tumor treating fields significantly increase survival rates in combination with adjuvant temozolomide (TMZ) in adult and GBM patients. Here, we report about feasibility and safety of treatment on a pediatric patient with diffuse midline glioma who is receiving TTFields therapy in combination with temozolomide.

## Introduction

High-grade gliomas (HGG) are aggressive brain tumors affecting both children and adults [1]. Treatment options are limited in pediatric HGG patients, and prognosis is fatal. Tumor treating fields (TTFields) are low-intensity, intermediate frequency (200 kHz) alternating electric fields used as a treatment for adult GBM [1].

Preclinical models demonstrated that TTFields inhibit proliferation of cancer cells by disruption of the mitotic spindle apparatus resulting in mitotic delay or cell death [2, 3]; furthermore, they impair DNA-repair-mechanisms and increase chemosensitivity through the induction of cell permeability as well as an increase of autophagy [4, 5]. Also, an influence on migratory capacity and antitumor immunity has been described [6]. Efficacy and safety of TTFields in adult ndGBM were examined in the phase 3 study EF-14 (*n* = 695), which demonstrated that the addition of TTFields to maintenance temozolomide (TMZ) significantly prolonged overall survival (OS) (20.9 vs 16.0, *p* < 0.001) and progression-free survival (PFS) (6.7 vs 4.0 months, *p* < 0.001) [7]. A subgroup analysis of the EF-14 patient cohort showed that patient compliance (therapy usage) was prognostic for improved survival [8]. Therefore, it is important to maintain high daily/monthly compliance to the TTFields treatment.

For the pediatric population, there are several reports on the use of TTFields. For instance, Branter J. et al. showed efficacy of TTFields on pediatric GBM, medulloblastoma and ependymoma cell lines in vitro [9]. Toledano H. et al. report on 5 pediatric patients (age 11.1–17.7 years at diagnosis) und conclude that TTFields is safe and feasible in children as young as 11 years [10]. Wölfl W. et al. presented 3 patients (age 7, 9, and 11 years) suffering from HGG [11]. Post-marketing surveillance data on TTFields collected for glioma patients < 18 years of age (*n* = 30) revealed no unexpected adverse events. The most frequently reported adverse events were skin reactions [12].

## Case report

**Fig. 1:**
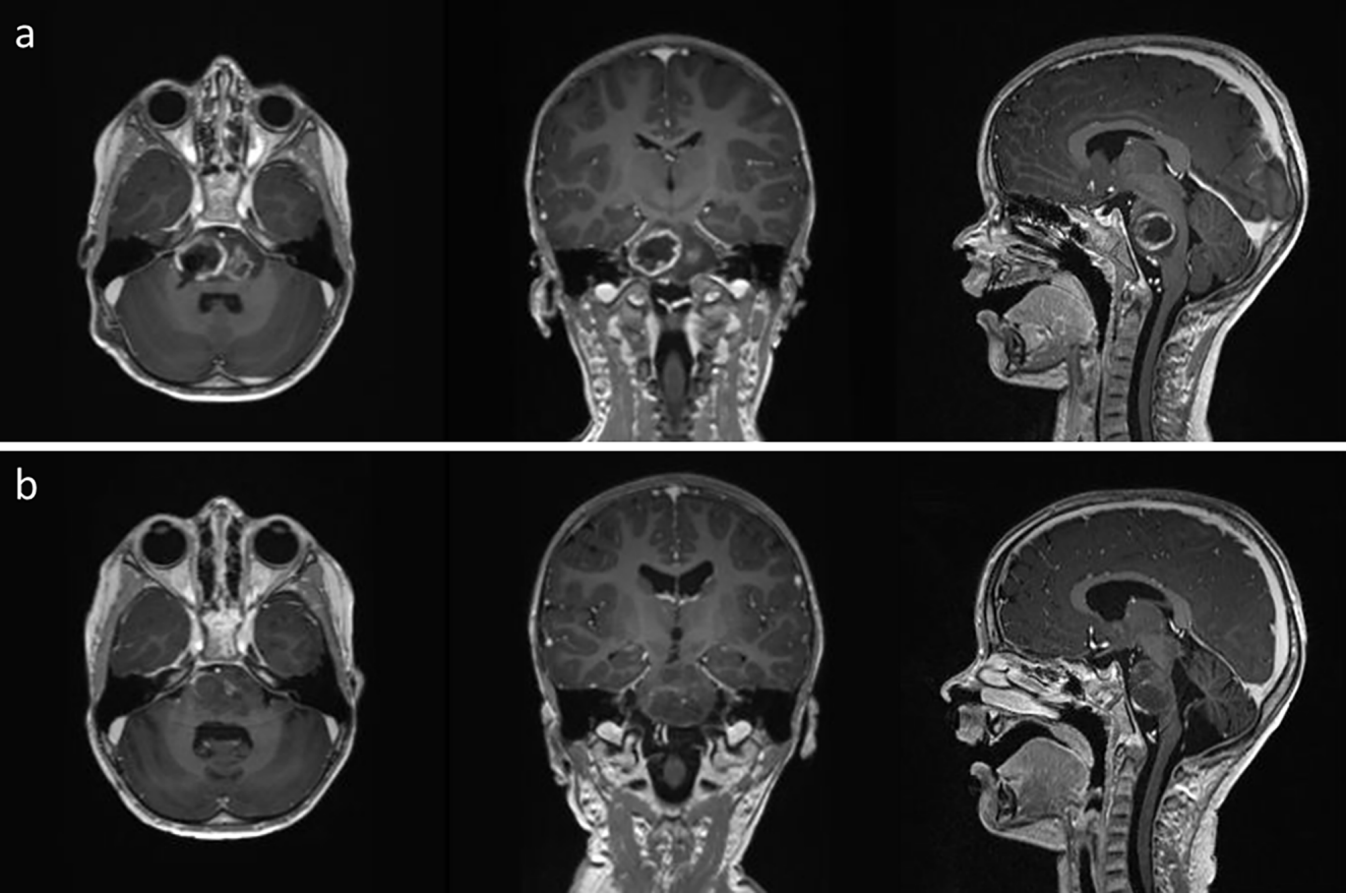
**a** Glioma with central necrotic area in the brainstem after stereotactic biopsy by an infratentorial approach. **b** The same lesion 1 year after biopsy followed by chemoradiation with TMZ maintenance and TTFields therapy.

**Fig. 2:**
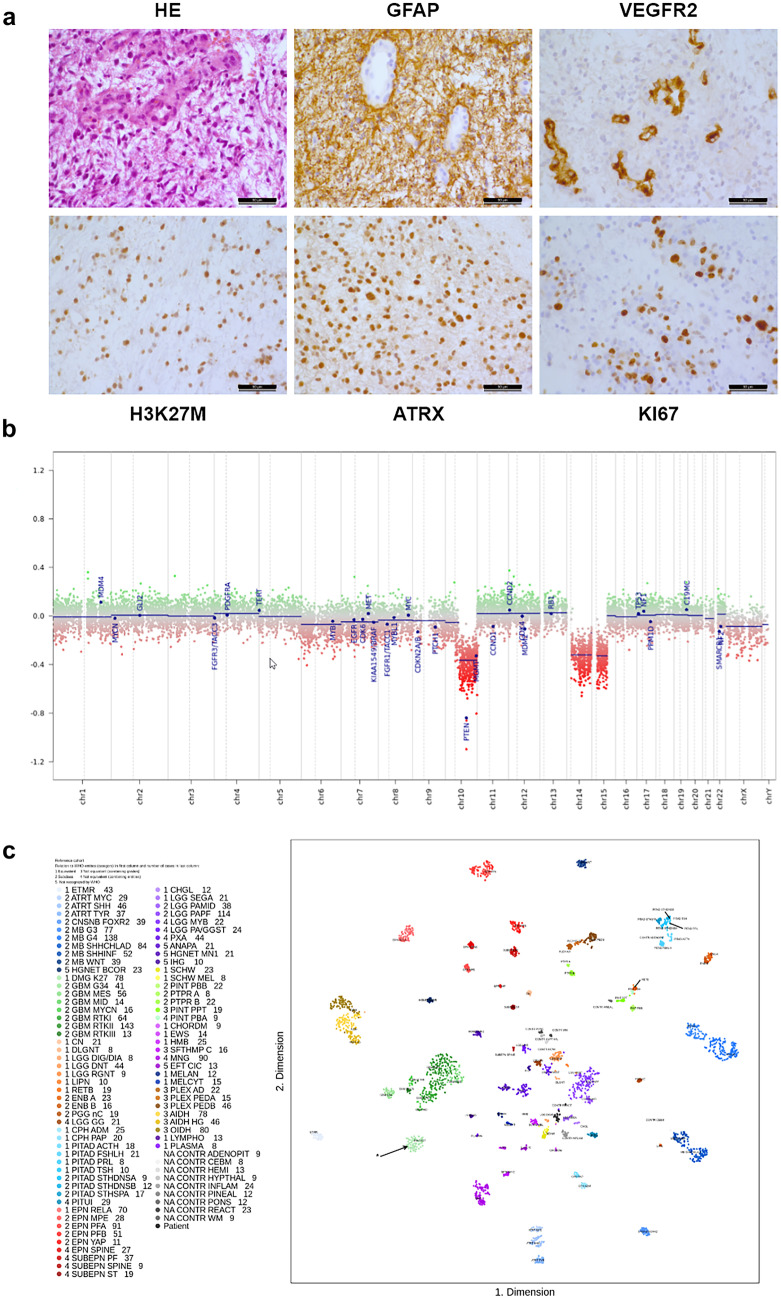
Histological features of a diffuse malignant pleomorphic glioma, H&E. Immunohistochemistry stained sections; tumor cells with GFAP expression and upregulation of VEGFR2. Nuclear H3K27M- and ATRX-Expression and high KI67 labeling (**a**). Copy number profile of diffuse midline glioma H3K27M mutant with a deletion of PTEN gene and especially a loss of chromosome 10 (**b**). t-SNE analysis showing DNA-methylation clustering of our case in the DMG K27 cohort (*n* = 78) (**c**). The data basis for the t-SNE evaluation are the 2801 methylome analyzes from Capper et. al. 2018. The 34,000 most variable positions are extracted from these and, together with the data record to be classified, these are reduced in their complexity using t-distributed neighbor embedding (t-SNE). The function was taken from the R — package R-t-SNE by Jesse Krijthe

The 3-year-old male patient presented with a tumor located pontine on the right occipital region (Fig. 1a). Stereotactic biopsy was performed, and with the brain tumor classifier employing genome wide DNA- methylation analysis [13], the diagnosis of a diffuse midline glioma H3K27M (calibrated Score 0.98) without MGMT-promoter methylation was obtained. Molecular sequencing detected a HIST1H3B mutation (H3.1 K27M) (Fig. 2). H3.1 and H3.2 K27M mutations are typically found in DIPGs and are associated with a better prognosis than the H3.3 K27M-wildtype [14–16].

The patient received radiochemotherapy with TMZ followed by TTFields therapy in combination with maintenance TMZ. Due to the location of the tumor, the infratentorial transducer array layout was used for TTFields application. The infratentorial layout was not yet available during the EF-14 study, and therefore not examined in a clinical setting, but a computer simulation-based study showed the feasibility of TTFields in treating tumors in infratentorial regions [17].

**Fig. 3:**
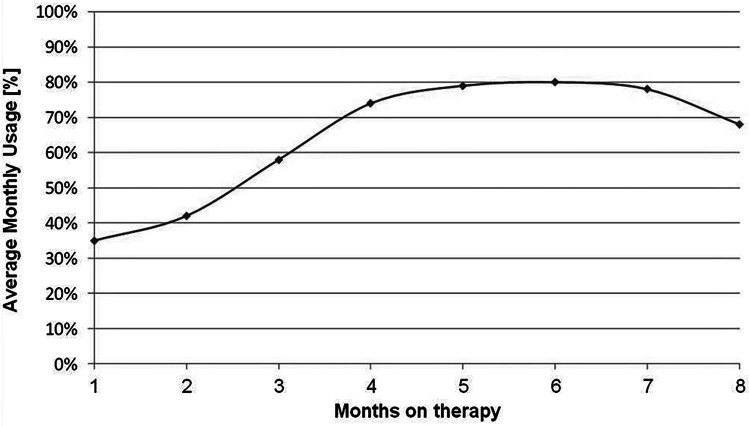
Average monthly TTFields usage rate in percentage over time. Therapy adherence to TTFields started at a low level and increased strongly within the first 4 months, showing that after an initial adaption period, high TTFields usage rates are also achievable in very young patients. Due to general decline in patient’s health state, the usage rate was reduced from month eight

Initially, the patient and his family needed time to get used to the TTFields therapy and due to the usage of TTFields therapy was comparably low during the first month. However, the TTFields usage rate improved every month from about 40% in the first 2 months to about 80% after 5 and 6 months (Fig. 3). After the initial time for adaptation to the therapy, the average usage rate in months 4 to 8 was 75.87%, which is above the independent prognostic threshold of 75% [11]. One year after the initial biopsy, the MRI showed distinct radiological response to the therapy (Fig. 1b). Unfortunately, 1 month later, the patient’s general health condition declined, causing reduced usage rates towards the end of TTFields therapy. These results demonstrate that even a very young patient is able to adapt to using the TTFields therapy. The patient was on TTFields therapy for almost 9 months and no therapy-related adverse events were observed.

## Discussion

Treatment options for pediatric patients diagnosed with HGG are limited and there is a high need to extend the therapeutic repertoire. TTFields are currently only approved for adult glioblastoma. However, there are various reports and ongoing trials on TTFields therapy applied to pediatric glioma patients [11, 18–20] (NCT03033992, NCT03128047). These reports are supported by post-marketing surveillance data that revealed no unexpected adverse events upon TTFields application in pediatric patients [21].

Based on available evidence, we decided in accordance with the family to apply TTFields therapy to a 3-year-old patient diagnosed with diffuse midline glioma. Before initiation of TTFields therapy, we had to determine whether it is possible to fit all four transducer arrays to the patient’s head as provided by the array layout. Due to the infratentorial layout provided for the patient, proper placement of the transducer arrays was feasible. In ongoing TTFields trials, the minimal head circumference for placement of transducer arrays was calculated to be 44 cm (NCT03033992, NCT03128047).

Another critical factor for TTFields treatment is the usage rate achieved by the patients. A subgroup analysis of the EF-14 phase 3 trial demonstrated that OS was extended when the usage rate was > 50%, and there was a trend for extended OS with higher usage rates. In addition, a monthly usage rate of ≥ 75% vs < 75% (TTFields plus TMZ arm) was an independent predictor of OS.

Because of these results, it is important to openly discuss the factor usage with patients/caregivers and provide guidance. It was shown before that it might take some time to adapt to the treatment [22]. In the case of a young child, depending on age, it might be more challenging to explain the necessity of a particular treatment scheme. However, as shown in Fig. 3, we found that the patient was quickly able to adapt to the treatment as shown by the steep increase in usage above the independent prognostic threshold of 75% [8]. This case clearly demonstrates that adherence to TTFields therapy can be continuously improved over time, even if the usage rate might be low in the beginning. When the usage rate is not satisfying initially, TTFields therapy does not have to be terminated, instead patient and caregiver should be encouraged and supported to improve therapy adherence over time as demonstrated in the presented case.

## Conclusion

In summary, TTFields therapy was feasible and safe in a pediatric patient 3 years of age at initiation of therapy. Safety and efficacy in pediatric patients are and will be further investigated in clinical trials**.**
